# The RNA-binding protein Puf5 contributes to buffering of mRNA upon chromatin-mediated changes in nascent transcription

**DOI:** 10.1242/jcs.259051

**Published:** 2021-08-05

**Authors:** David Z. Kochan, Julia S. P. Mawer, Jennifer Massen, Kiril Tishinov, Swati Parekh, Martin Graef, Anne Spang, Peter Tessarz

**Affiliations:** 1 Max Planck Research Group ‘Chromatin and Ageing’, Max Planck Institute for Biology of Ageing, Joseph-Stelzmann-Str. 9b, 50931 Cologne, Germany; 2 Biozentrum, University of Basel, Klingelbergstrasse 50/70, 4056 Basel, Switzerland; 3 Max Planck Research Group ‘Autophagy and Cellular Ageing’, Max Planck Institute for Biology of Ageing, Joseph-Stelzmann-Str. 9b, 50931 Cologne, Germany; 4 Cologne Excellence Cluster on Stress Responses in Ageing-Associated Diseases (CECAD), University of Cologne, Joseph-Stelzmann-Str. 26, 50931 Cologne, Germany

**Keywords:** Chromatin, Transcription, Post-transcriptional buffering, Histone modification, RNA-binding protein

## Abstract

Gene expression involves regulation of chromatin structure and transcription, as well as processing of the transcribed mRNA. While there are feedback mechanisms, it is not clear whether these include crosstalk between chromatin architecture and mRNA decay. To address this, we performed a genome-wide genetic screen using a *Saccharomyces cerevisiae* strain harbouring the H3K56A mutation, which is known to perturb chromatin structure and nascent transcription. We identified Puf5 (also known as Mpt5) as essential in an H3K56A background. Depletion of Puf5 in this background leads to downregulation of Puf5 targets. We suggest that Puf5 plays a role in post-transcriptional buffering of mRNAs, and support this by transcriptional shutoff experiments in which Puf5 mRNA targets are degraded slower in H3K56A cells compared to wild-type cells. Finally, we show that post-transcriptional buffering of Puf5 targets is widespread and does not occur only in an H3K56A mutant, but also in an H3K4R background, which leads to a global increase in nascent transcription. Our data suggest that Puf5 determines the fate of its mRNA targets in a context-dependent manner acting as an mRNA surveillance hub balancing deregulated nascent transcription to maintain physiological mRNA levels.

## Abstract (precis)

**Summary:** Identification of a pathway involving the yeast RNA-binding protein Puf5 that ensures maintenance of correct mRNA levels when chromatin alterations lead to changes in nascent transcription.

## INTRODUCTION

The life of an individual mRNA starts with its synthesis from the DNA template. This process is catalysed by RNA polymerase II and is strongly impacted on by the underlying architecture and dynamics of chromatin. Indeed, the packaging of DNA into nucleosomes affects all stages of transcription, from transcription factor binding to initiation and elongation (Workman and Kingston, 1998). Nucleosomes are subject to a vast array of post-translational modifications (Tessarz and Kouzarides, 2014). These regulate (in-)directly the accessibility of the underlying DNA and, thus, the efficiency of transcription. For instance, acetylation of lysine side chains in histones neutralizes its positive charge and weakens the histone–DNA interaction to promote transcription. Although we have a solid understanding of the molecular events governing transcription, we still have limited insight into the regulation of mRNA decay. This is largely because standard RNA-seq experiments capture the steady-state levels of RNA and thereby do not shed light on its dynamic lifecycle (Nikopoulou et al., 2019). Recent studies have addressed synthesis and degradation rates (Chan et al., 2018; Miller et al., 2011; Munchel et al., 2011). These approaches have helped to reveal the dynamic lifecycle of mRNA, showing that the average mRNA half-life is only a few minutes. This highlights the importance of mRNA degradation pathways in maintaining cellular homeostasis.

Degradation of mRNA is initiated by the removal of the poly(A) tail, which in yeast is mainly catalysed by the cytoplasmic Ccr4–Not complex (Collart, 2016). Subsequently, mRNAs can either be degraded from the 3′ end by the exosome complex or, following mRNA decapping, from the 5′ end by the cytoplasmic exonuclease, Xrn1 (Muhlrad and Parker, 1994). Targeting of mRNAs to the Ccr4–Not complex is mediated by several RNA binding proteins, including proteins of the Pumilio family of proteins (Pufs) (Nishanth and Simon, 2020). Different Pufs recognize Pumilio-response elements (PREs) of different sizes and have overlapping, but also distinct, target RNAs. Puf5 (also known as Mpt5), for instance, can bind ∼16% of all yeast mRNAs, a network that is partly shared with Puf3 and Puf4 (Lapointe et al., 2017).

In this study, we set out to address whether there is a connection between chromatin dynamics and the complex post-transcriptional network of RNA-binding proteins. Could global changes in the underlying histone modification pattern, such as during cancer development or ageing, influence the post-transcriptional life of an mRNA? The rationale for this study comes from observations in mutants affecting acetylation of histone H3K56. Acetylation at this site is important to allow access to chromatin after DNA damage, enhance histone turnover at transcriptionally active chromatin, and to promote nucleosome assembly during S phase (Kaplan et al., 2008; Li et al., 2008; Topal et al., 2019; Wurtele et al., 2012). Despite this important role in shaping chromatin, mutation of H3K56 to arginine (R) only mildly affects the steady-state transcriptome (Rege et al., 2015; Topal et al., 2019). Intriguingly though, genome-wide nascent transcription is reduced in these mutants (Topal et al., 2019), suggesting that post-transcriptional buffering of mRNA levels must exist. Such a phenomenon has been implicated in earlier work using an RNA polymerase II variant with decreased elongation speed (Sun et al., 2012). Using a genome-wide genetic screen in *Saccharomyces cerevisiae*, we identify *puf5*Δ as synthetically lethal with H3K56A, indicating that Puf5 might work as a central player in this buffering system through its targeting of downregulated nascent transcripts in an H3K56A background. Depletion of Puf5 in this background leads to the downregulation of ribosomal protein genes, suggesting that the reason for synthetic lethality is a decrease in translation. We further identify mutations in H3K4 that are also genetically linked to Puf5, indicating that the phenomenon of buffering mRNAs upon chromatin-mediated transcriptional change is widespread. Strikingly, in the case of H3K4R, nascent transcription is upregulated, suggesting a context-specific activity of Puf5. Such a post-transcriptional buffering system would constitute another regulatory layer in gene expression to ensure cellular homeostasis.

## RESULTS

### A high-throughput screen identifies synthetic lethality in an H3K56A *puf5*Δ double mutant

**Fig. 1.**
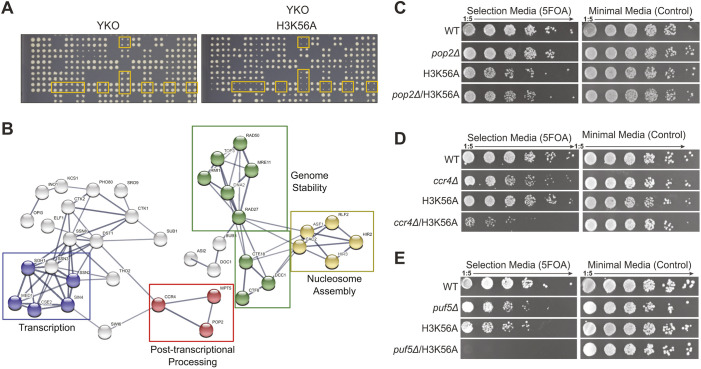
**Identification of lethal genetic interaction between *puf5*Δ and H3K56A.** (A) Representative SGA plates highlighting several synthetic genetic interactions between H3K56A and yeast deletion strains (YKO) (right plate). Every mutant is represented in four replicates. (B) STRING network representation of protein complexes identified to be genetically linked to H3K56A. The GO term for the individual complexes are highlighted by different colours. For details on gene names and GO annotations, please refer to Table S1. (C–E) Validation of synthetic interactions of the SGA in W303 using a plasmid shuffle system to introduce H3K56A into the indicated genomic deletions of *POP2* (C), *CCR4* (D) and *PUF5* (E).

The observation that, in an H3K56A yeast mutant, a reduction in nascent transcription does not lead to a reduction in steady-state levels of mRNA, indicates the presence of post-transcriptional buffering of gene expression. We propose that mutations that disrupt this buffering process may lead to lethality. To this end, a high-throughput synthetic genetic array (SGA) screen was performed. The H3K56A mutant was used as a query strain, and screened against the entire yeast knockout (YKO) and Decreased Abundancy by mRNA Perturbation (DAmP) collections (Breslow et al., 2008; Giaever et al., 2002) (Fig. 1A). In total, 101 synthetic interactions were identified (Table S1). These included several metabolic complexes, genes required for genome stability and members of the nucleosome assembly machinery, as well as components of the Mediator complex (Fig. 1B). Interestingly, constituents of the Ccr4–Not poly-deadenylation complex (Ccr4, Pop2 and Puf5) were also identified (Fig. 1B). As the Ccr4–Not poly-deadenylation complex is known to function in the processing of mRNA (Collart, 2016), its components could be ideal candidates for a post-transcriptional buffering system of gene expression. To verify the results from the screen, *pop2*, *ccr4* and *puf5* deletions were generated in a W303 background that allows for the introduction of histone mutations by plasmid shuffle (Tessarz et al., 2014) (Table S1). Using this system, the synthetic genetic interaction of H3K56A with *pop2*Δ could not be reproduced (Fig. 1C), while a growth defect was observed for H3K56A with *ccr4*Δ (Fig. 1D). The H3K56A *puf5*Δ double mutant, however, was confirmed to be synthetically lethal (Fig. 1E).

Despite Puf5 being an adapter protein for the core Ccr4–Not machinery, these results suggest that the genetic interaction between H3K56A and Puf5 is at least in part independent of Ccr4–Not. In support of this, Puf5 has recently been identified as central to regulating mRNA stability in the absence of the Ccr4–Not complex (Wang et al., 2018). Taken together, using a synthetic genetic screen, we have identified a member of the Puf protein family to be synthetically lethal with an alanine substitution in histone H3 at K56. The identification of a gene known to be involved in post-transcriptional control of mRNA levels supports our initial hypothesis that the decreased level of nascent mRNA in an H3K56A mutant might be protected from degradation and thus post-transcriptionally buffered to ensure homeostasis of gene expression and cell survival.

### Cytoplasmic localization and RNA binding are essential for Puf5 function in an H3K56A background

**Fig. 2.**
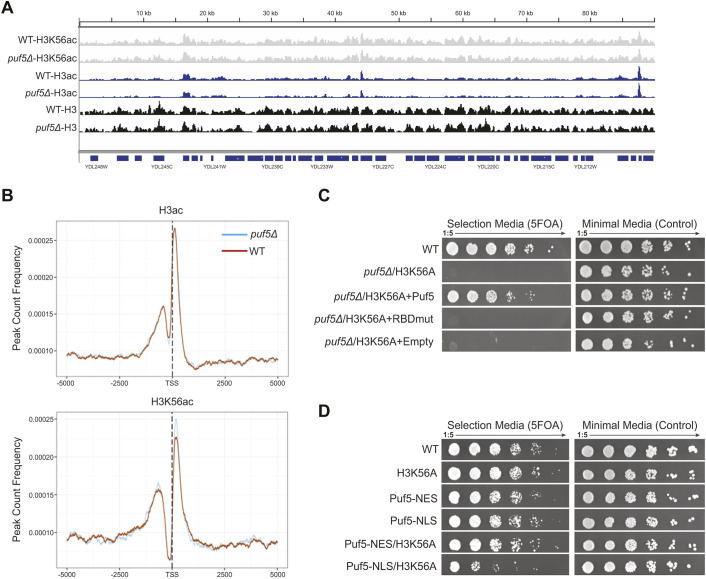
**Puf5 function is downstream of chromatin, localised in the cytoplasm and requires RNA-binding ability.** (A) Representative genome browser track of 100 kb of chromosome IV showing similar occupancy of pan-ac H3, H3K56ac and core H3 in wild-type (WT) and *puf5*Δ cells. (B) Metaplot over the transcription start site (TSS) +/− 5,000 bp comparing intensities of pan-ac H3 and H3K56ac in WT and *puf5*Δ strains. (C) Complementation assay using a Puf5 mutant unable to bind RNA (RBDmut; S454A, N455A; Traven et al., 2010). (D) Complementation assay using either cytoplasmic (NES)- or nuclear (NLS)-localised Puf5.

Several mRNA targets of Puf5 code for proteins involved in the maintenance of chromatin architecture (Lapointe et al., 2017; Wilinski et al., 2015). Therefore, *puf5*Δ could result in changes in chromatin structure that become lethal upon loss of acetylated H3K56 (H3K56ac). To determine whether a *puf5*Δ mutant has aberrant chromatin structure, chromatin immunoprecipitation (ChIP)-seq was used to assess genome-wide deposition of H3, acetylated H3 (pan-acH3, recognizes acetylated K9, K14, K18, K23, K27 of H3) and H3K56ac. No differences in the genome-wide levels of H3, pan-acH3 and H3K56ac were observed in a *puf5*Δ mutant (Fig. 2A). There were also no significant changes in pan-acH3 and H3K56ac levels at transcription start sites (Fig. 2B), indicating that *puf5*Δ does not have an impact on overall chromatin architecture. These ChIP-seq results suggest that Puf5 functions downstream of H3K56 and support a post-transcriptional role for this protein.

Puf5 is an RNA-binding protein. As we hypothesise that its function in an H3K56A background is to protect target mRNAs from premature degradation, the RNA-binding domain would be expected to be essential for this function. To test this, a Puf5 RNA-binding domain mutant (RBDmut) (S454A, N455A; Traven et al., 2010), was generated and used to complement the H3K56A *puf5*Δ double mutant. As expected, wild-type *PUF5* was able to fully rescue the H3K56A *puf5*Δ lethality, while the Puf5 RBDmut protein was not, confirming that Puf5's mRNA-binding ability is essential for H3K56A mutant survival (Fig. 2C). Importantly, Puf5 was expressed to similar levels independent of whether it had or did not have the RBD mutation (Fig. S1A). Puf5 is localized in the cytoplasm and nucleus. To test whether cellular localization plays an important role for the potential buffering of mRNAs, we tagged Puf5 with either a nuclear localization signal (NLS) (Traven et al., 2010) or a nuclear export signal (NES). To evaluate the efficiency of the tag, we also added a GFP tag and monitored Puf5 localization under the microscope, and found targeting was consistent with correct targeting of the protein to the respective cellular localisation (Fig. S1B). Interestingly, whereas Puf5 localized to the cytoplasm complemented a Puf5 deletion, nuclear Puf5 only partially complemented a Puf5 deletion (Fig. 2D), indicating that Puf5 has to be cytoplasmic to buffer the effects of an H3K56A mutation. Together, these data suggest that Puf5 functions downstream of H3K56A, in the cytoplasm, and requires its RNA-binding ability.

### Rapid degradation of Puf5 in an H3K56A background leads to downregulation of ribosomal protein genes

To identify genes and pathways that rely on the potential buffering effect of Puf5 in the H3K56A background, we used an auxin-induced degradation (AID) system (Tanaka et al., 2015) to quickly degrade Puf5 in the presence of the histone mutation using doxycycline and auxin (Dox/Aux; Fig. S2A). This system combines transcription shut-off through a Tet-off promoter with auxin-induced degradation that is mediated by an AID tag. However, this system led to a slight overexpression of Puf5. Therefore, we pre-treated cells with low doses of Dox overnight, which decreased Puf5 transcripts, returning them to wild-type levels (Fig. S2B). More importantly, in the presence of Dox/Aux, this system recapitulated the synthetic defect between H3K56A and *puf5*Δ in liquid culture and on agar plates (Fig. S2C,D), while in their absence, the strain showed a similar expression programme to an H3K56A strain (Fig. S2E).

**Fig. 3.**
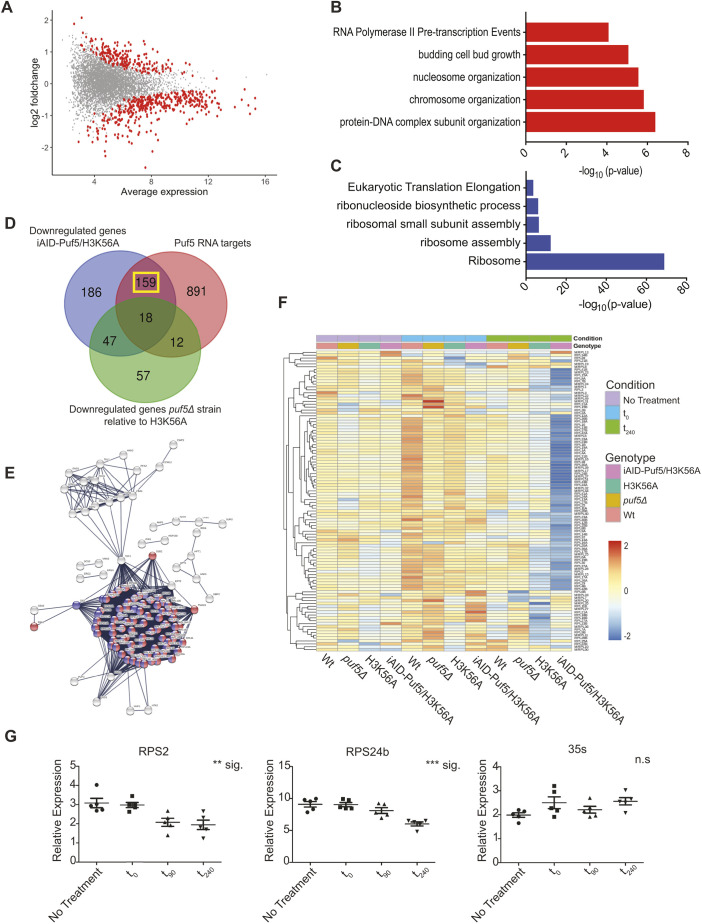
**Rapid degradation of Puf5 reveals ribosomal protein genes as targets for Puf5 buffering.** (A) MA plot comparing H3K56A strains before and after depletion of Puf5. Significantly deregulated genes are highlighted in red. RNA-seq was performed using a 3′-end RNA-seq library preparation protocol with unique molecular identifiers (UMIs) for proper quantification of mRNAs (*n*=5). (B,C) Top GO terms for (B) upregulated and (C) downregulated genes upon rapid depletion of Puf5 in an H3K56A background. (D) Overlap of downregulated genes upon Puf5 depletion and its targets, as identified by eCLIP, and differentially regulated genes between *puf5*Δ and H3K56A. (E) STRING representation of the 159 genes that overlap in (D; yellow box) reveals ribosomal proteins (red and blue) as the main target genes for buffering. For details on the 159 genes highlighted in this network, please refer to Table S2. (F) Mean expression level of ribosomal protein genes across the various genetic backgrounds and conditions tested for by RNA-seq. (G) RT-qPCR of selected ribosomal protein genes at indicated time points during the depletion of Puf5 in an H3K56A background. 35S rRNA transcription was measured to assess the impact on the nascent transcription of ribosomal DNA. All values are relative to actin. Error bars are s.e.m. **P<0.01; ***P<0.001; n.s., not significant (one-way ANOVA without post hoc test).

As additional controls, we performed RNA-seq on wild-type, H3K56A and *puf5*Δ strains. In line with previously published data, we did not observe strong differential gene expression between a wild-type and H3K56A strain (Fig. S2F), while a *puf5*Δ deletion showed a significant change in the gene expression programme (Fig. S2F). Comparing the individual strains after addition of Dox/Aux, it is obvious that only degradation of Puf5 led to significant changes in the transcriptome, indicating that the combined Dox/Aux treatment only affected the degron strain (Fig. S2G). Acute depletion of Puf5 in the H3K56A background led to upregulation of 238 genes, while 410 genes were significantly downregulated (Fig. S3A). Upregulated genes were enriched for functions in chromatin organization-, cell wall- and cell cycle-related terms (Fig. 3A,B), which might be a potential compensatory response as H3K56A *puf5*Δ cells become very large during the arrest. Strikingly, downregulated genes were strongly enriched for ribosome-related terms (Fig. 3C). To identify mRNAs that might represent Puf5-buffered mRNAs in the H3K56A background, we intersected all downregulated genes upon Puf5 depletion in the H3K56A background with (1) genes downregulated between *puf5*Δ and H3K56A and (2) direct Puf5 targets as identified by eCLIP (Wilinski et al., 2015) (Fig. 3D). We then focused on the 159 mRNAs that were direct targets of Puf5 and were specifically downregulated in the double mutant strain (yellow box, Fig. 3D), which largely consisted of ribosomal protein genes as well as ribosome assembly and translation initiation factors (Fig. 3E,F). These results suggest that the synthetic growth defect of Puf5 depletion in the H3K56A background is, at least to a large extent, caused by downregulation of ribosomal protein genes. To assess the specificity of the downregulation for ribosomal protein transcripts in the double mutant, we also analysed rDNA transcription. Importantly, while we could confirm downregulation of ribosomal protein genes, nascent 35S rRNA transcription was not affected by Puf5 depletion (Fig. 3G), demonstrating that the downregulation of ribosomal protein transcripts is a specific and direct consequence of Puf5 depletion, arguing against an unspecific cellular stress response. As downregulation of ribosomal protein genes might directly impact cell cycle progression (Bernstein et al., 2007), we wanted to investigated whether the observed synthetic interaction between Puf5 and H3K56A might be due to a cell cycle arrest rather than death. To address this, we plated cells 4 h after Puf5 depletion in the presence or absence of Dox/Aux. While on Dox/Aux, cells harbouring the Puf5 degron did not grow, in their absence, cells recovered and resumed growth (Fig. S2H). These data indicated that the depletion leads to an arrest during the cell cycle. However, cell cycle analysis by fluorescence-activated cell sorting (FACS) indicated that cells did not arrest in any specific cell cycle stage (Fig. S2I).

Interestingly, Puf5 shares transcripts of ribosomal protein genes as targets in common with Puf3 and Puf4 but not with Puf1, Puf2 or Puf6 (Lapointe et al., 2017). This led us to hypothesize that deletion of Puf3 and Puf4 might also have a growth defect when combined with H3K56A. We systematically deleted *puf1–puf4* and *puf6* in combination with H3K56A. *puf1*, *puf2* and *puf6* deletions did not result in any genetic interaction, but *puf3*Δ and *puf4*Δ showed mild growth phenotypes (Fig. S3), suggesting a partial overlap of substrates with Puf5. Importantly, Puf3 and Puf4 only share a fraction of these transcripts with Puf5 (Lapointe et al., 2017), which might explain the much weaker phenotype observed for the deletion of these two Puf proteins.

### Puf5 targets are degraded more slowly in an H3K56A background

**Fig. 4.**
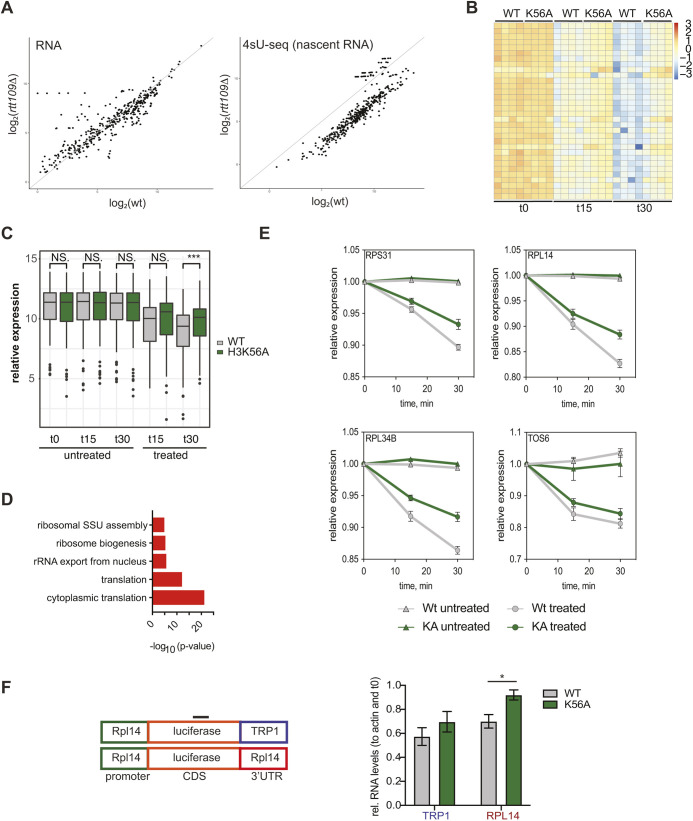
**Puf5-buffered mRNAs are degraded more slowly in an H3K56A background.** (A) Comparison between bulk (steady-state) mRNA levels in wild-type (WT) and *rtt109*Δ mutants as assessed by nascent RNA (4sU-seq) levels in the same strains. Plotted are mRNAs identified to be downregulated upon Puf5 depletion in an H3K56A background (see Fig. 3). Data was taken from Topal et al. (2019). (B) Heatmap of mRNAs with a significantly altered degradation rate between WT and H3K56A upon transcription shutoff using thiolutin. Cells were grown to mid-log phase before transcription was halted by addition of thiolutin and RNA was extracted at the indicated time points. (C) Boxplot highlighting the differences in mRNA levels for all genes with differentially altered degradation rates. The box represents the 25–75th percentiles, and the median is indicated. The whiskers show the values within 1.5× of the interquartile range from the upper and lower quartiles. *n*=4. ***P<0.001; n.s., not significant (unpaired two-tailed *t*-test). (D) GO terms associated with mRNAs identified in B. (E) Individual examples of altered degradation rate comparing WT and H3K56A backgrounds. Results are mean±s.d. (*n*=4). (F) Reporter assay using the indicated reporter constructs. RNA was measured 30 min after addition of thiolutin. Results are mean±s.e.m. (*n*=5). *P<0.05 (based on an unpaired two-sided *t*-test).

To directly test the hypothesis of post-transcriptional buffering of reduced nascent transcription by Puf5, we asked whether the mRNAs identified as downregulated upon depletion of Puf5 in the H3K56A background are (1) deregulated on the level of nascent transcription and (2) are indeed buffered, that is degraded more slowly in the H3K56A mutant in order to maintain a comparable level of these transcripts to that seen in wild type. To address the first point, we made use of a previously published large dataset comparing nascent transcription in a variety of mutants unable to acetylate H3K56 (Topal et al., 2019) and intersected this dataset with our RNA-seq results (Fig. 4A). In particular, we used bulk RNA-seq as well as nascent RNA-seq (4sU-seq) datasets from wild-type and *rtt109*Δ strains, which lack H3K56 acetylation (GSE125843; Topal et al., 2019). Indeed, while the steady-state mRNA levels remain stable between wild type and *rtt109Δ*, the level for nascent transcripts decreased significantly in the histone acetyltransferase deletion strain (Fig. 4A).

This result supports the idea that Puf5 might buffer at least a subset of reduced nascent transcripts. Recent data suggested a very simple way for cells to control the amount of mRNA at the post-transcriptional level that involves the general 5′-3′ exonuclease Xrn1. The model proposed suggests a simple feedback mechanism based on Xrn1 levels that might be sufficient to buffer mRNA on a global scale (Sun et al., 2013). An overall decrease in transcription would lead to lower Xrn1 levels (on mRNA and, subsequently, protein levels), which in turn would lead to lower mRNA degradation. An increase in transcription would result in an increase in Xrn1 levels and thus, an increase in degradation of all mRNAs. However, no evidence of altered Xrn1 levels were found in our RNA-seq data for all experimental conditions tested (Fig. S4A). Additionally, we observed only a mild genetic interaction between H3K56A and *xrn1*Δ (Fig. S4B). An overexpression of XRN1 in H3K56A/*puf5Δ* background only led to a minor rescue of the lethality (Fig. S4C) suggesting that Xrn1 plays some role in the observed H3K56A/*puf5*Δ phenotype. However, as in the case for Ccr4, the *puf5* lethality in the H3K56A background is, at least to a substantial part, independent of Xrn1. Finally, to directly measure the difference in degradation rates between a wild-type and H3K56A strain, we performed transcriptional shut-off experiments (Collier, 2008). Thiolutin was used to block transcription in logarithmically growing yeast and RNA was isolated after 0, 15 and 30 min to assess the level of degradation. Subsequently, we generated 3′-end RNA-seq libraries with UMIs to identify mRNAs that were differentially degraded in a WT and H3K56A background. While thiolutin has been reported to have an impact on mRNA stability on its own (Pelechano and Pérez-Ortín, 2008), the overall effect of thiolutin was comparable in both strain backgrounds (Fig. S4D). Using this approach, we identified mRNAs that showed a significantly altered degradation pattern, in particular mRNAs that were stabilized in an H3K56A background (Fig. 4B). A boxplot showing the difference in the mean expression of these mRNAs confirmed the overall stabilization (Fig. 4C). Given that the differences in degradation rates are subtle over the relatively short time of the experiment and are thus difficult to state based on the stringent criteria of an RNA-seq analysis, we only identified ∼50 mRNAs. However, the trend in overall levels indicates that there might be many more transcripts that follow the observed pattern (Fig. S4E). In line with the idea that Puf5 might be able to buffer expression of its target genes in an H3K56A background, differentially degraded mRNAs are strongly enriched for transcripts encoding ribosomal proteins (Fig. 4D). Finally, plotting the expression levels over time demonstrate an overall slower degradation kinetics for ribosomal protein-encoding mRNAs *RPS32*, *RPL34B* and *RPL14*, whereas no difference was observed for *TOS6* (Fig. 4E). To address more specifically the role of Puf5 in this process, we generated reporter constructs that differed only in the 3′UTR, and thus the Puf5-binding region. We used the TRP1 3′UTR as control and RPL14 3′UTR as a known Puf5 target that was also degraded significantly slower in an H3K56A background (Fig. 4E). Transcriptional shutoffs were performed in WT and H3K56A for both constructs and the remaining transcript levels were measured after 30 min. The reporter construct containing the RPL14 3′UTR was degraded more slowly in an H3K56A background (Fig. 4F). These results confirm that the 3′UTR is important for determining mRNA stability and support the idea of Puf5 as a mediator for this stability.

### Post-transcriptional buffering by Puf5 is more widespread and also protects against upregulated nascent transcription

**Fig. 5.**
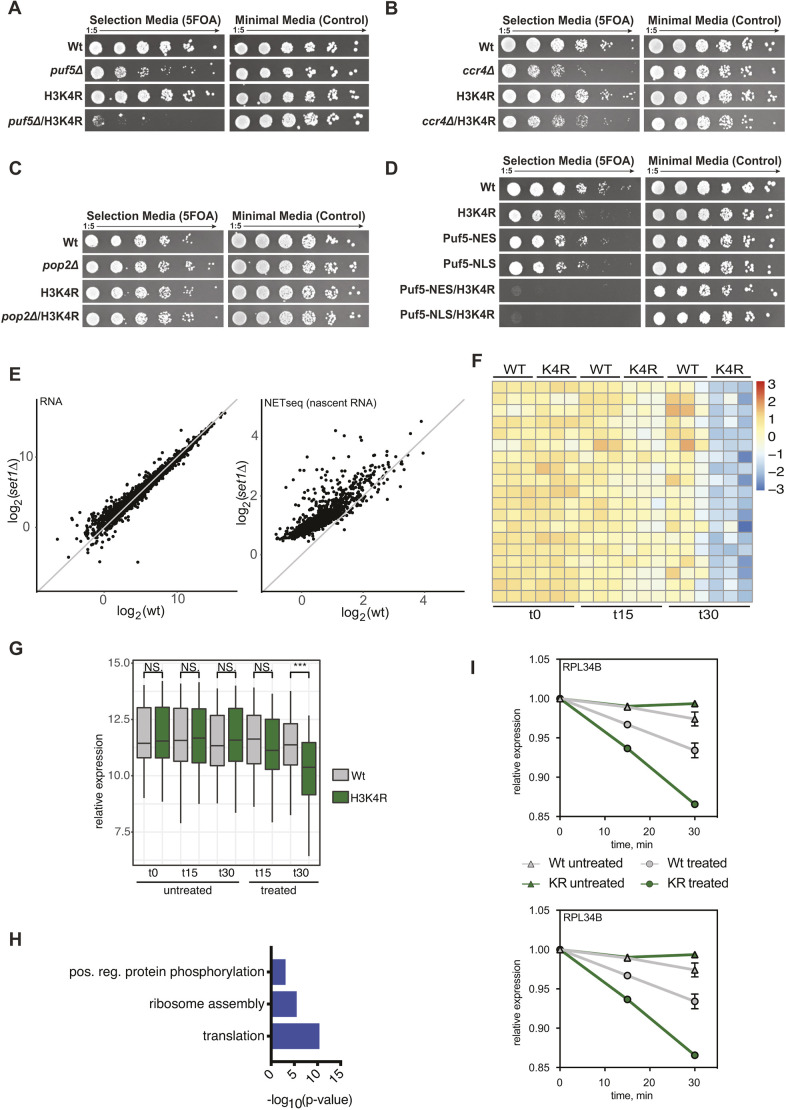
**Deletion of PUF5 is synthetically sick with H3K4R.** (A) Spot test showing synthetic sickness between H3K4R and *puf5*Δ. No synthetic genetic interaction was seen by combining an H3K4R mutant with deletions in (B) *ccr4* and (C) *pop2*. (D) Complementation assay to test for a sufficiency of Puf5 being recruited to cytoplasm (NES) or nucleus (NLS). (E) Comparison of RNA steady-state and nascent RNA levels between deletion of the H3K4 methyltransferase, Set1, and wild-type (WT) cells. (F) Heatmap of mRNAs with a significantly altered degradation rate between WT and H3K4R upon transcription shutoff using thiolutin. Cells were grown to mid-log phase before transcription was halted by addition of thiolutin and RNA was extracted at the indicated time points. (G) Boxplot highlighting the differences in mRNA levels for all genes with differentially altered degradation rates. The box represents the 25–75th percentiles, and the median is indicated. The whiskers show the values within 1.5× of the interquartile range from the upper and lower quartiles. *n*=4. ***P<0.001; n.s., not significant (unpaired two-tailed *t*-test). (H) GO terms associated with mRNAs identified in F. (I) Individual examples of altered degradation rate comparing WT and H3K4R backgrounds based on normalised RNA-seq values. Results are mean±s.d. (*n*=4).

Another interesting aspect to explore was how widespread this phenomenon of mRNA buffering is. To start to address this question, we performed a mini-screen with histone mutations that change amino acids known to be involved in either DNA replication (H4 K5 and K12) or transcription (H3 K4, K16, K36 and K79) (Fig. S5A–D). Of these mutations, H3K4R showed a strong synthetic phenotype in combination with *puf5*Δ (Fig. 5A) that was comparable to the one observed with H3K56A, thus indicating that a potential Puf5 buffering effect might be more widespread. No genetic interaction with *ccr4*Δ or *pop2*Δ was observed (Fig. 5B,C), indicating that the role of Puf5 is independent of its classical function as an adaptor for the Ccr4–Not complex – similar to its role in the H3K56A background. The lack of synthetic interaction between H3K4R and *ccr4*Δ or *pop2*Δ might also be due to the fact that the H3K56A impact seems to be stronger than that of H3K4R. However, we observed two very obvious differences compared to the H3K56A *puf5*Δ genetic interaction. First, the cytoplasmic localisation of Puf5 is not sufficient to complement an H3K4R *puf5*Δ double mutant (Fig. 5D). Second, the potential targets of Puf5 in an H3K4R background are not transcripts that stem from downregulated nascent transcription. We compared bulk RNA-seq (GSE52086; Martín et al., 2014) and nascent RNA (NET-seq) data (GSE25107; Churchman and Weissman, 2011) for a deletion of *SET1*. One should note that one limitation in this analysis is the fact that this original NET-seq data did not contain spike-in as compared to the H3K56-related experiments*. SET1* is the sole methyltransferase in yeast responsible for methylating H3K4 (Briggs et al., 2001; Santos-Rosa et al., 2002), thus mimicking an H3K4R mutation. In *set1*Δ, nascent RNA was upregulated compared to wild-type levels, whereas steady-state mRNA levels were largely indistinguishable from those in wild-type cells (Fig. 5E). This observation suggests that Puf5 might make context-specific decisions with respect to degradation or stabilisation of mRNA. This idea is supported by the observation that an H3K56Q strain – which mimics an Hst3/4 double knockout and thus, should lead to higher nascent transcription – is also synthetically lethal with *puf5* (Fig. S5E). To test this hypothesis more directly, we performed transcriptional shutoff experiments in an H3K4R background. As was the case for the H3K56A background, the genome-wide impact of thiolutin was comparable between WT and H3K4R (Fig. S5F). In contrast to what was seen with H3K56A, we identified mRNAs that were degraded faster in the H3K4R background compared to in wild type (Fig. 5F). All mRNAs identified were previously shown to be Puf5 targets (Wilinski et al., 2015). A boxplot showing the difference in the mean expression of these mRNAs confirmed the overall faster degradation (Fig. 5G). Despite not being significant, the genome-wide trend suggests that there are potentially many more transcripts that are degraded faster in an H3K4R background compared to in wild type (Fig. S5G). Again, gene ontology (GO) enrichment revealed that the transcripts with a significantly different degradation rate were enriched for proteins involved in translation (Fig. 5H). While RPS31 and RPL34B were stabilized in an H3K56A background, these transcripts were degraded faster in the H3K4R background (Fig. 5I), supporting the idea that transcripts can be either stabilized or degraded depending on the level of nascent transcription.

## DISCUSSION

Using a genome-wide genetic screen, we uncovered a post-transcriptional buffering system that can counter defects in nascent transcription to maintain the steady-state level of mRNAs. This is the first report that connects chromatin-mediated changes to nascent transcription with a post-transcriptional surveillance system. Strong circumstantial evidence presented here implicates Puf5 in the buffering of its target mRNAs upon deregulated nascent transcription; however, one limitation of our study is the fact that we were not able to devise an experiment that would prove the direct involvement of Puf5 in buffering. A potential avenue might be the use of the auxin-mediated degron system in combination with transcriptional shut-off experiments. However, such an experimental approach will be technically very challenging as the timing of Puf5 degradation and/or the transcriptional shut-off will be critical and the read-out will depend solely on RNA-seq analysis. It is important to note in this regard though that previous observations have linked Puf proteins to context-specific decisions, whether to degrade or stabilize transcripts in different environmental conditions (Lee and Tu, 2015; Wang et al., 2018). Future work will have to address how Puf proteins are able to make these complex decisions. Importantly, Puf proteins only bind a select number of target mRNAs through their RBD and the corresponding PRE and thus, will not be a general buffering system as has been suggested for Xrn1 (Haimovich et al., 2013; Miller et al., 2011; Sun et al., 2013), which can have a global impact on gene expression. Rather, the data presented here, together with data published previously (Lee and Tu, 2015; Wang et al., 2018), suggest that the buffering mediated by Puf proteins is only required under specific conditions and balances only a subset of transcripts that are defined by the PRE.

Notably, a connection between the RNA surveillance machinery and chromatin architecture has been made before. Deletions of *rrp6*, a component of the nuclear exosome leads to an increase of ∼1000 mRNAs, in addition to many cryptic unstable transcripts (CUTs) (Rege et al., 2015). An additional deletion of *rtt109* reduces the levels of these two classes of RNAs again. Subsequent ChIP-seq revealed that these observations can at least in part be explained by a reduced recruitment of RNA polymerase II to open reading frames and CUTs in the absence of H3K56 acetylation. Similar to the concept presented here though, the nuclear exosome somehow senses a surplus in transcription and degrades the excess amount of transcripts (Rege et al., 2015). However, it is not clear whether faulty mRNAs are produced or how the nuclear exosome can identify an excess of transcription.

The two residues whose mutation was found to have synthetic interactions with *puf5*Δ are of H3K4 and H3K56. Interestingly, both residues and their modification have recently been implicated in playing a major role in buffering gene expression during DNA replication, in which the transcript number of a given gene remains the same although the underlying DNA sequence is duplicated (Voichek et al., 2016, 2018). It is tempting to speculate that Puf5 might be involved in this process too. Unfortunately, it is difficult to evaluate this in the context provided here, as most transcripts detected as being downregulated upon Puf5 depletion in the H3K56A background were originally excluded from the cell cycle buffered transcripts since ribosomal protein genes strongly change in abundance during the cell cycle (Voichek et al., 2016). In addition, it is important to note that the phenomenon of replication-specific transcriptional buffering was not seen using a different approach (Topal et al., 2019), thus future experiments have to address whether and how mRNA buffering occurs upon DNA replication in *S. cerevisiae*.

A post-transcriptional buffering system would constitute an important system to maintain transcriptional stability under conditions in which transcriptional rates are impacted, for example, upon exposure to UV irradiation (Andrade-Lima et al., 2015). In addition, during ageing, many post-translational modifications on histones become deregulated and change in abundance. The levels of H3K56ac, for instance, are already strongly decreased in yeast cells that underwent six or seven divisions compared to juvenile yeast cells (Dang et al., 2009). A buffering system might be able to compensate for this loss of histone acetylation. This speculation is supported by the following observations that (1) overexpression of Puf5 prolongs replicative lifespan (Kaeberlein and Guarente, 2002; Kaeberlein et al., 2004; Kennedy et al., 1995), whereas (2) deletion of Puf5 strongly decreases replicative lifespan (Kaeberlein and Kennedy, 2005).

The family of Pumilio RNA-binding proteins is highly conserved among most eukaryotes (Wang et al., 2018). It will be exciting to see whether this phenomenon of post-transcriptional buffering extends not only to other chromatin-mediated effects and cellular states, but also to other organisms. The increased availability of techniques to measure nascent transcription will allow us to identify conditions and settings that impact nascent transcription based on environmental cues, which are subsequently buffered to sustain the mRNA pool and maintain cellular homeostasis.

## MATERIALS AND METHODS

### Strains, plasmids and reagents

Genotypes of strains and yeast plasmids used in this work are listed in Table S3 and S4. All chemicals used in this study were purchased analytical grade from either Sigma-Aldrich or Carl Roth except for the following: Drop Out Mix for yeast synthetic medium (SD) was from US Biological Life Sciences (D9543-01); 5-FOA was bought from Cayman Chemical (17318); Ampure XP beads were from Beckman Coulter (A63881); Protein-G coupled dynabeads from Thermo Fisher Scientific (10009D) and Thiolutin from Abcam (ab143556). Secondary antibodies against rabbit (7074S) and mouse (7076S) IgG coupled to horseradish peroxidase (HRP) were purchased from Cell Signaling. Antibodies against H3K56ac were from Active Motif (39281), G6PDH (A9521) and Myc (MABE282) from Sigma, iAID-tag (M214-3) from MBL International and β-actin (GTX109639) from GeneTex. Antibody dilutions were as suggested by the manufacturer (Table S12).

### Yeast protocols

If not stated otherwise, all strains used were derivatives of W303. Integrations and deletions were performed using one-step PCR-based methods (Janke et al., 2004; Longtine et al., 1998; Tanaka et al., 2015). Yeast were grown in YPD medium. Protein was extracted from yeast using sodium hydroxide lysis and TCA precipitation as previously described (Knop et al., 1999). RNA was extracted using the hot phenol approach (Schmitt et al., 1990). For spot tests, cells were grown over-night, diluted to an optical density at 595 nm (OD_595_)=1 and 5-fold serially diluted.

### Auxin-mediated degradation of Puf5

Single yeast colonies were picked and inoculated overnight in 5 ml of YPD medium at 30°C. The next day, cultures were diluted in 20 ml of YPD medium to an OD_595_ of ∼0.15 and grown at 30°C until an OD_595_ of 0.6–0.8. From each cell culture, 10 ml were collected for RNA extraction and 1 ml of cell culture was diluted in 4 ml of YPD medium containing 0.25 μg/ml of Dox and incubated overnight at 30°C. The following day, the overnight cultures were diluted in 40 ml of YPD medium containing 0.25 μg/ml of Dox to an OD_595_ of 0.2 and incubated at 30°C to an OD_595_ of 0.6–0.8. From each cell culture, 10 ml were then collected for RNA extraction and the remaining cell culture was diluted in 40 ml of YPD medium containing 40 μg/ml of Dox and 1 mM Aux to an OD_595_ of ∼0.2. The cell cultures were then further incubated at 30°C and additional samples were taken at 90 and 240 min.

### Transcriptional shutoff

Cells were grown in 100 ml of YPD (30°C and 200 rpm rotation) to mid-log phase (OD_600nm_ 0.5–0.6). Cultures were then split and either DMSO or 3 μg/ml of thiolutin (Abcam ab143556, reconstituted in DMSO) were added. Cells were sampled at time-points of 0, 15, 30 and 60 min. Total RNA was extracted using the hot phenol method (Schmitt et al., 1990). Reverse transcription was performed using random hexamers with 0.5–1 μg of total RNA. Quantitative (q)PCR was performed and the ΔΔC_t_ method was used to measure relative expression. 18S rRNA and time-point 0 were used as reference. RNA-seq libraries were prepared as described below.

### 3′-RNA sequencing and analysis

Bulk 3′ RNA-seq libraries were prepared following the SCRB-seq protocol (Bagnoli et al., 2018) using five biological replicates per genotype and time point. A detailed protocol is available on request from the corresponding author. Briefly, 50 ng per sample was reversed transcribed using a polyT_30_ oligonucleotide containing a 8 bp barcode for later identification and a unique molecular identifier (UMI). Reverse transcription used the template switching. After the reverse transcription step, all reverse transcribed samples were pooled into a single 2 ml Eppendorf tube and DNA was purified with Ampure XP (Beckman, #A63881) beads at 1:1 (v/v). Purified DNA was eluted in 17 μl H_2_O and treated with exonuclease (New England Biolabs, #M0568). Subsequently, cDNA was amplified and the final PCR product was purified with Ampure XP beads at a volume to volume ratio of 0.8. Finally, 0.8 ng cDNA was tagmented in five replicates. The quality of library was assessed on an Agilent Tapestation and sequenced on an Illumina HiSeq4000 in a 2×75 bp mode to ∼1 million fragments per sample. The sequenced reads were processed using zUMIs (version 2.0.6) (Parekh et al., 2018 preprint) with STAR (version 2.6.1a) (Dobin et al., 2013), samtools (version 1.9) (Li et al., 2009) and featureCounts (Liao et al., 2014) from Rsubread (version 1.32.4). The reads were mapped to the yeast genome (R64) with the Ensembl annotation version 91. The genes were filtered using the ‘filterByExpr’ function of edgeR (Robinson et al., 2010) with the min.count=5. The differential gene expression analysis was carried out using the limma-voom (Law et al., 2014; Ritchie et al., 2015) approach at the adjusted *P*-value of 0.05. Obtained sets of differentially expressed genes were further analysed, for example, through gene ontology (GO) enrichment analysis. Differential gene expression analysis for all conditions can be found in Tables S5–S11. GO term enrichment was performed using Metascape (Zhou et al., 2019) and interaction networks were generated using STRING (Szklarczyk et al., 2019).

### ChIP-sequencing and analysis

Chromatin immunoprecipitation and library production was performed as previously described (Tessarz et al., 2014). The fastq reads were mapped to yeast genome (R64) using bowtie2 (Langmead and Salzberg, 2012) local alignment and duplicates were then removed using MarkDuplicates program of Picard Tools. The peaks were called using MACS2 (version 2.1.1.20160309) (Gaspar, 2018 preprint), with settings –nomodel, --extsize 150, -B -q 0.05 --keep-dup 1. The peaks were annotated using the ChIPseeker package (Yu et al., 2015). The differential analysis between wild type and *puf5D* was carried out using edgeR (Robinson et al., 2010). The normalising factors were calculated using ‘RLE’ method within ‘calcNormFactors’, the tagwise dispersion trend was estimated using the default parameters in ‘estimateDisp’ function and a generalised linear model was then fitted on the data using the ‘glmQLFit’ function in robust mode. The genome binning for differential analysis was performed using csaw (Lun and Smyth, 2016) with the binsize of 1000 and global window filtering with the minimum enrichment of 2 over background.

### Yeast cell cycle analysis by FACS

Cell culture containing 2×10^7^ cells was spun down (1000 ***g*** for 5 min), resuspended in 0.5 ml water, sonicated for 30 s in an ultrasonic bath and ethanol was added to a 70% final volume. The samples were agitated at an Eppendorf Thermomixer Compact at top speed for 10 min, then left overnight at 4°C. Cells were flash spun, resuspended in 0.5 ml water, flash spun again (1000 ***g*** for 5 min), and resuspended in 0.5 ml RNase A (2 mg/ml in 50 mM Tris-HCl, pH 8.0, with DNase inactivated by prior addition by incubating the solution at 95°C for 20 min). The samples were shaken at 37°C for 2 h at an Eppendorf Thermomixer Compact at top speed and then pelleted (1000 ***g*** for 5 min). Pellets were washed with 0.5 ml water and resuspended in freshly prepared pepsin solution (5 mg/ml in 50 mM HCl). The samples were shaken at 37°C for 20 min at an Eppendorf Thermomixer Compact at top speed, pelleted (1000 ***g*** for 5 min), washed with 0.5 ml water, resuspended in 0.2 ml 50 mM Tris-HCl, pH 7.5 and sonicated in an ultrasonic cleaning bath for 30 s. The suspensions were passed through a 35-μm nylon cell-strainer (Falcon), propidium iodide (PI) was added to 50 μg/ml final from a 500 μg/ml stock solution in water. The samples were vortexed and placed in the dark. Aliquots (25 µl) of the samples with PI were diluted with 950 µl 50 mM Tris-HCl, pH 7.5 and analysed immediately at a BD FACS CantoII Analyzer at the low speed flow setting. PI detection was carried out with the 488 nm laser and the 670 long pass filter. A total of 10,000 or 50,000 events were analysed.

## Supplementary Material

Supplementary information
