# Supplementary material for: The RNA-binding protein Puf5 contributes to buffering of mRNA upon chromatin-mediated changes in nascent transcription
Source: J Cell Sci. 2021 Aug 5;134(15):jcs259051. doi: 10.1242/jcs.259051 (PMC8353526; doi:10.1242/jcs.259051)
Supplement: Supplementary information [file joces-134-259051-s1.pdf]

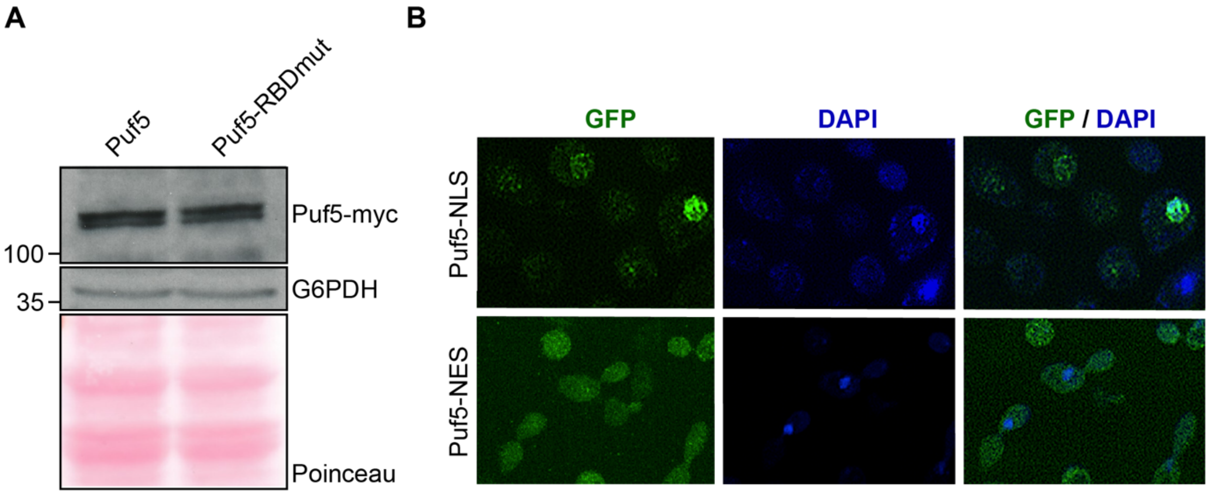

Fig S1. A) Total levels of Puf5-myc fused to NES or NLS respectively as measured using anti-myc western blotting. Glucose-6-phosphate dehydrogenase (G6PDH) and Poinceau served as loading control. B) Subcellular localisation of Puf5-GFP fused to NES or NLS, respectively. DAPI served as counterstain for the nucleus.

Journal of Cell Science • Supplementary information

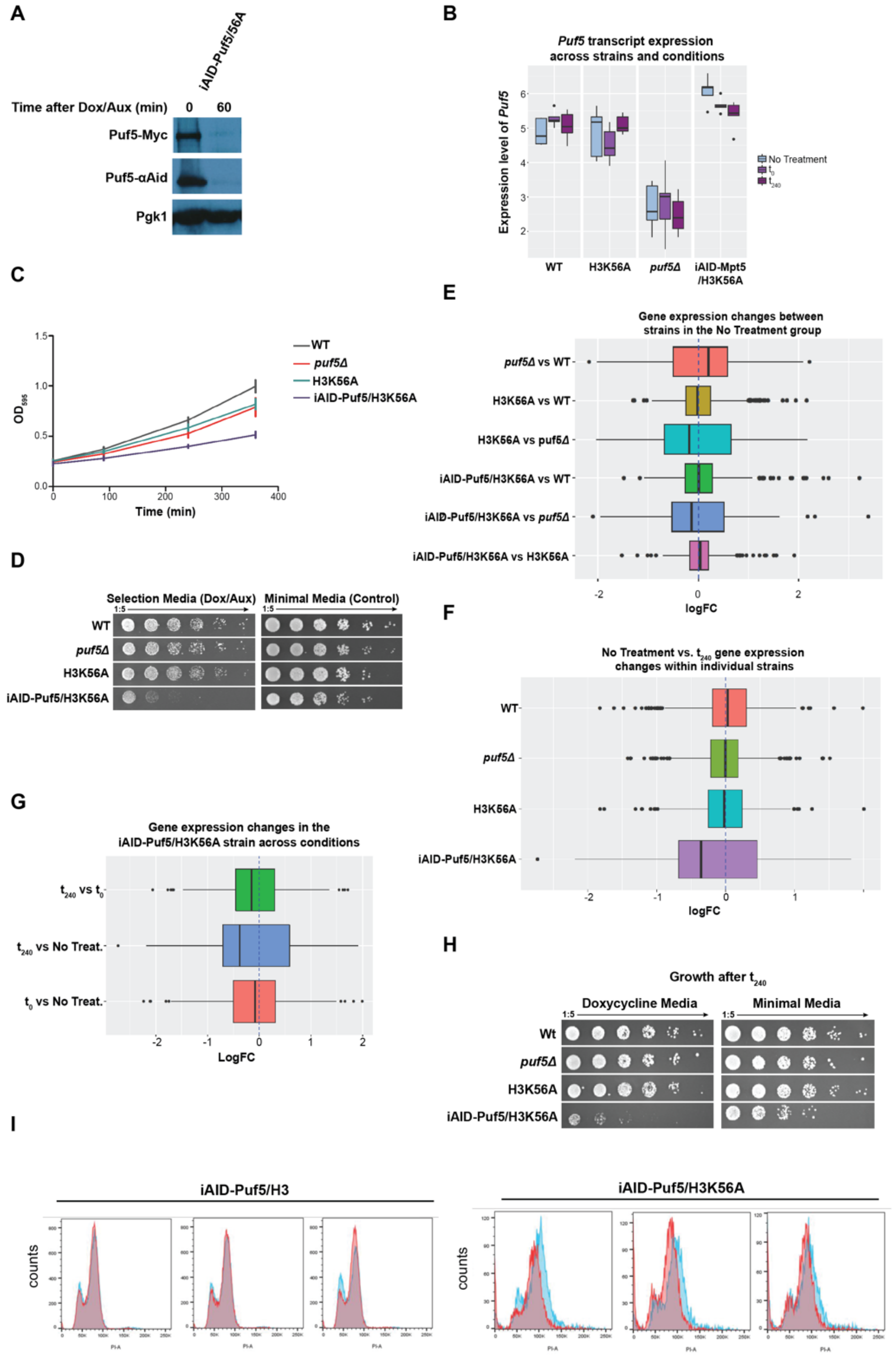

Fig. S2. A) Western blot against N-terminal 3xAID-tag and C-terminal myc-tagged Puf5 before and 60min after 40Î¼g/ml and 1mM auxin addition. B) Expression level of Puf5 in the individual strains used in the RNA-seq upon Puf5 depletion, with or without Dox/Aux addition. Impact of Dox/Aux addition on the growth of yeast in liquid (C) and solid medium (D). E) Differential gene expression changes comparing the individual strains used in the RNA-seq experiment to assess downregulation upon rapid Puf5 depletion. (F) Differential gene expression changes upon treatment of Dox/Aux in the individual strains. (G) Differential gene expression changes upon Puf5 depletion in an H3K56A background. H) After 240min of Aux/Dox treatment, cells were either plated on SDC with/or without Dox/ Aux. I) Cell cycle analysis of Puf5-depleted yeast cultures expressing the H3 wild-type protein or the K56A mutant version. Logarithmically growing cells before (blue) or after 4 h depletion of Puf5 with Aux/Dox (red) were fixed and analyzed by FACS.

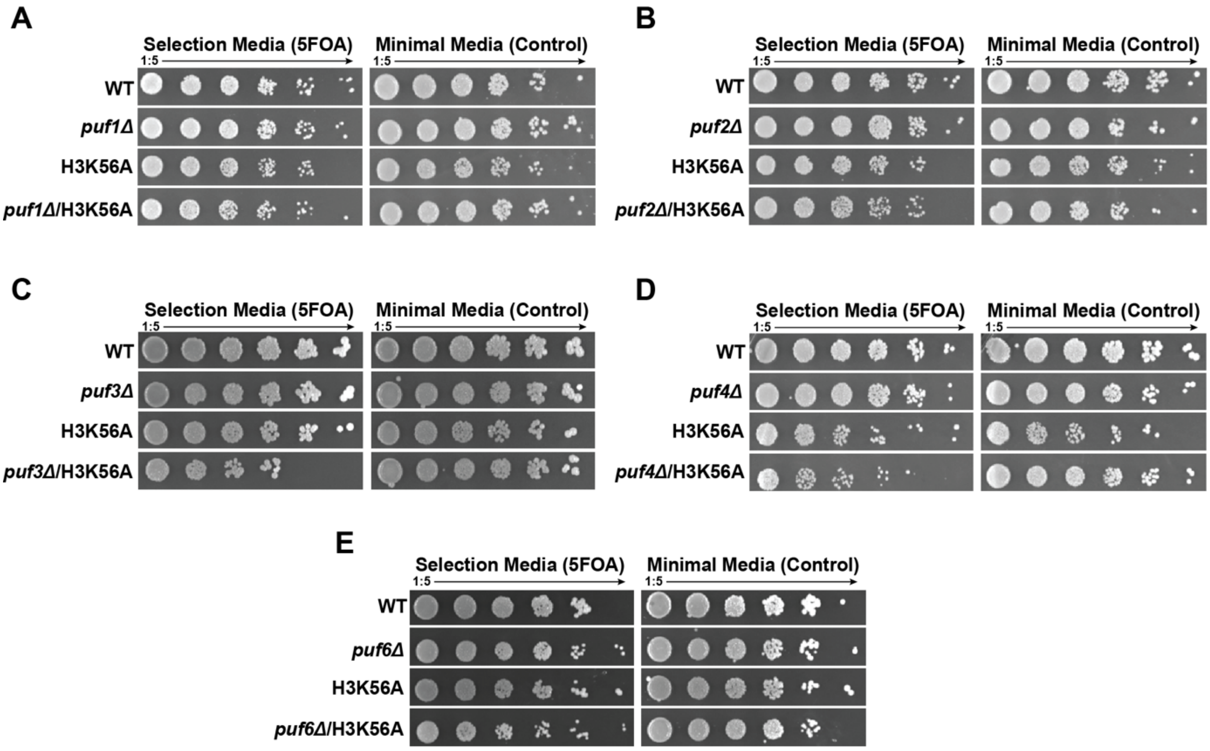

Fig. S3. H3K56A shows very mild genetic interactions with PUF3 and PUF4. Spot tests using the histone shuffle strain in combination with A) *puf1Δ*, B) *puf2Δ*, C) *puf3Δ*, D) *puf4Δ* and E) *puf6Δ* deletions.

Journal of Cell Science • Supplementary information

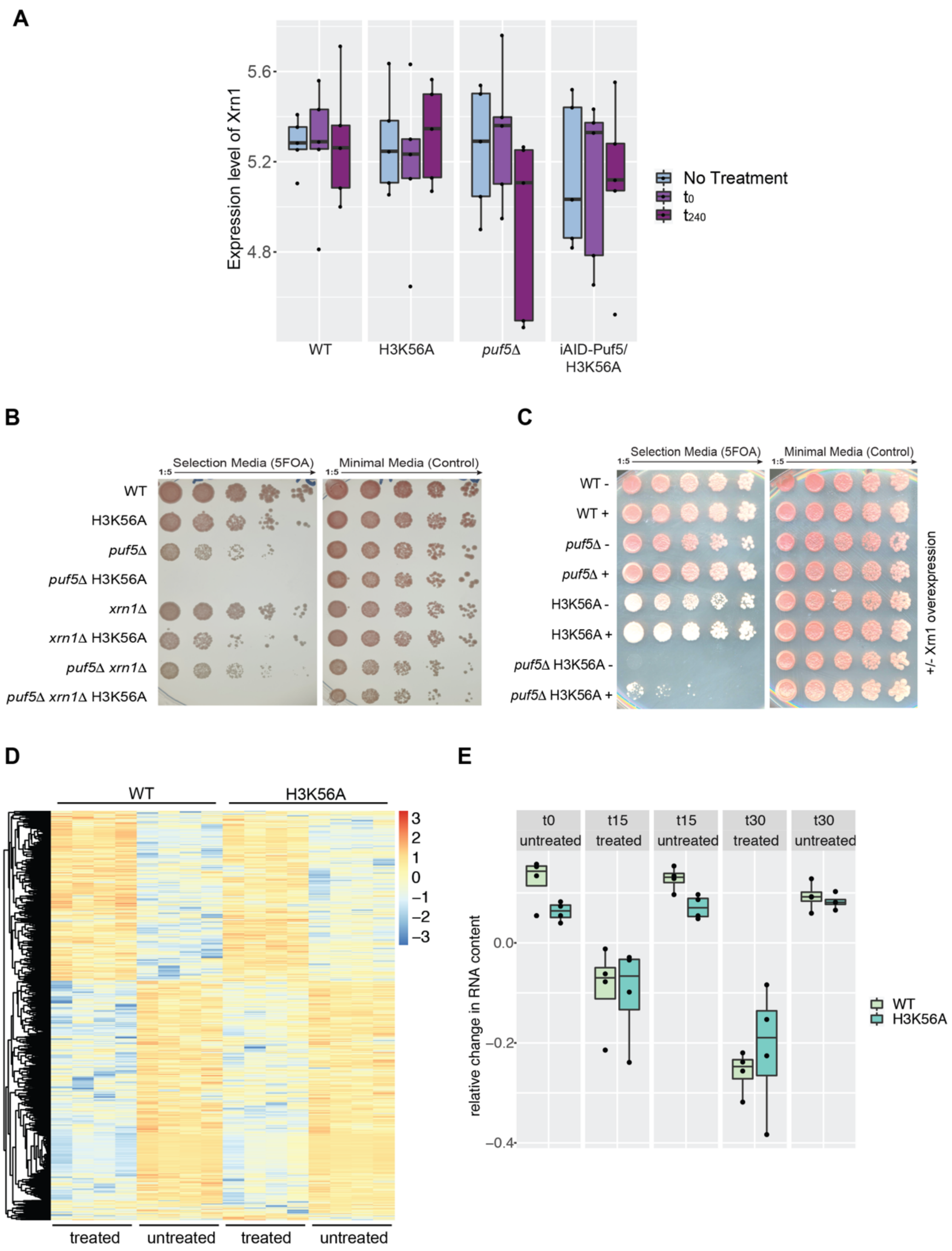

Fig. S4. Xrn1 plays a minor role in buffering against H3K56A-mediated de-regulation of nascent transcription. A) Xrn1 mRNA levels across the different conditions tested upon degradation of Puf5, including the Xrn1 levels in the H3K56A background. B) Spottest examining the impact of *xrn1* deletions on *puf5* and/or H3K56A mutations. C) Over-expression of Xrn1 in *puf5Δ*, H3K56A and the double mutant. D) Genome/wide impact of thiolutin treatment in an H3K56A background illustrated as heatmap depicting the transcriptional changes monitored genome-wide across the different conditions sampled upon thiolutin addition. E) Overview of the relative changes in mRNA level across the different treatments and time points.

Journal of Cell Science • Supplementary information

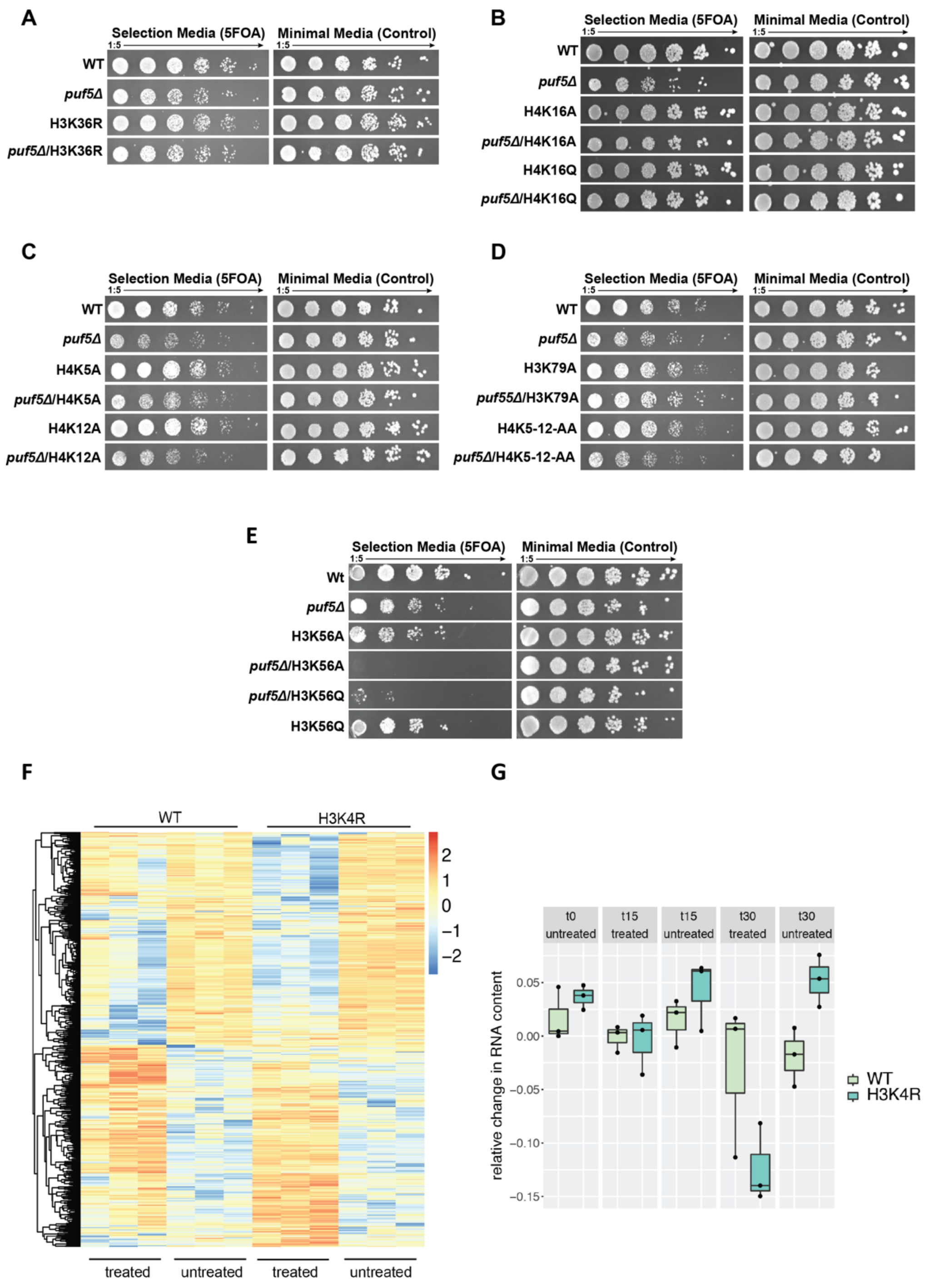

Fig. S5. Puf5 is synthetically lethal with an H3K4R mutation. A-D) Various histone mutations of sites involved in transcription and replication were shuffled into the *puf5* deletion strain. E) Puf5 is synthetically lethal with mutations in H3K56. Serial dilutions (1:5) were spotted on minimal media with or without 5FOA to test for abiliy to lose a wildtype histone plasmid. F) Genome/wide impact of thiolutin treatment in an H3K4R background illustrated as heatmap depicting the transcriptional changes monitored genome-wide across the different conditions sampled upon thiolutin addition. E) Overview of the relative changes in mRNA level across the different treatments and time points.

Table S1. Genes identified to be synthetically lethal/sick with an H3K56A mutation (relates to Figure 1)

Click here to download Table S1

Table S2. Overlap in gene list of mRNAs identified in Puf5 CLIP and upon degradation of Puf5 in H3K56A (relates to Figure 3D).

Click here to download Table S2

Table S3. Yeast strains used in this study.

Click here to download Table S3

Table S4. Plasmids used in this study.

Click here to download Table S4

Table S5. Differential mRNA levels in H3K56A iAID-Puf5 before Auxin addition.

Click here to download Table S5

Table S6. Differential mRNA levels in H3K56A iAID-Puf5 at different time points of Auxin and Doxycycline addition.

Click here to download Table S6

Table S7. Differential mRNA levels in all strains tested for H3K56A iAID-Puf5 before Auxin and Doxycycline addition.

Click here to download Table S7

Table S8. Differential mRNA levels in H3K56A iAID-Puf5 at time point t=240.

Click here to download Table S8

Journal of Cell Science • Supplementary information

Table S9. Differential mRNA levels in the H3K56A iAID-Puf5 experiment comparing t=0 (before auxin) and t=240

Click here to download Table S9

Table S10. Differential mRNA levels in H3K4R iAID-Puf5 comparing t=0 (before Auxin) and t=240.

Click here to download Table S10

Table S11. Differential mRNA levels in H3K4R iAID-Puf5 comparing WT and H3K4R at individual time points.

Click here to download Table S11

Table S12. Antibody dilutions.

Click here to download Table S12

Journal of Cell Science • Supplementary information
